# Graphene–ferroelectric metadevices for nonvolatile memory and reconfigurable logic-gate operations

**DOI:** 10.1038/ncomms10429

**Published:** 2016-01-27

**Authors:** Woo Young Kim, Hyeon-Don Kim, Teun-Teun Kim, Hyun-Sung Park, Kanghee Lee, Hyun Joo Choi, Seung Hoon Lee, Jaehyeon Son, Namkyoo Park, Bumki Min

**Affiliations:** 1 Department of Mechanical Engineering, Korea Advanced Institute of Science and Technology(KAIST), Daejeon 305-701, Republic of Korea; 2 Metamaterial Research Centre, School of Physics and Astronomy, University of Birmingham, Birmingham B15 2TT, UK; 3 Photonic Systems Laboratory, School of EECS, Seoul National University, Seoul 151-744, Republic of Korea

## Abstract

Memory metamaterials are artificial media that sustain transformed electromagnetic properties without persistent external stimuli. Previous memory metamaterials were realized with phase-change materials, such as vanadium dioxide or chalcogenide glasses, which exhibit memory behaviour with respect to electrically/optically induced thermal stimuli. However, they require a thermally isolated environment for longer retention or strong optical pump for phase-change. Here we demonstrate electrically programmable nonvolatile memory metadevices realised by the hybridization of graphene, a ferroelectric and meta-atoms/meta-molecules, and extend the concept further to establish reconfigurable logic-gate metadevices. For a memory metadevice having a single electrical input, amplitude, phase and even the polarization multi-states were clearly distinguishable with a retention time of over 10 years at room temperature. Furthermore, logic-gate functionalities were demonstrated with reconfigurable logic-gate metadevices having two electrical inputs, with each connected to separate ferroelectric layers that act as the multi-level controller for the doping level of the sandwiched graphene layer.

## Abstract (web summary)

Metamaterial memory devices often require a large stimulus to switch states or suffer from poor thermal stability. Here, the authors fabricate a terahertz metadevice driven by ferroelectric and graphene layers, and obtain multiple level memory devices stable at room temperature.

## Graphical abstract

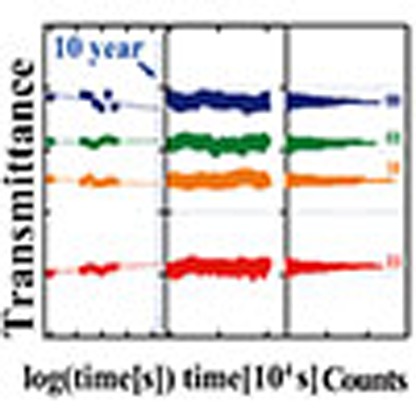

Metamaterials are artificial media that exhibit unusual electromagnetic properties such as anomalous refraction^1^,^2^,^3^,^4^,^5^, invisible cloak^6^,^7^ and strong chirality^8^,^9^. Light–matter interaction can be dramatically intensified with the use of electromagnetic responses around the resonance of specifically designed meta-atoms (MAs). Most of the wave properties, such as amplitude^10^, phase^11^ and polarization states^12^, and even the direction of light^13^ can be manipulated by the use of metamaterials or metasurfaces. Furthermore, variability in light–matter interaction is manifested through the external application of electrical^10^,^14^, mechanical^15^, optical^16^,^17^ and thermal^18^ stimuli when they are hybridized with natural active media. However, application of persistent stimuli is still required to sustain the transformed metamaterial properties unless there are memory functionalities. In this aspect, memory metamaterials are unique because long-lasting modification of the effective properties is possible even with the application of impulsive stimuli. This may lead to additional saving of energy resources that may otherwise have been used in sustaining the transformed properties. Previous memory metamaterials were implemented with natural memory media, such as phase-change materials^19^,^20^,^21^,^22^,^23^,^24^,^25^, that show memory behaviour with respect to electrically or optically induced thermal stimuli. The memory metamaterials have shown the possibility for unique performances, such as dynamic resonance tuning, multi-level data storage and bi-directionality^14^,^19^,^20^,^21^,^22^,^23^,^24^,^25^. However, they require a thermally isolated environment to obtain a longer retention of transformed properties or a strong optical pump for phase-change.

Here, we demonstrate electrically programmable nonvolatile memory metadevices operating at room temperature that are made possible by the hybridization of graphene, a ferroelectric and MAs/meta-molecules. The doping level of graphene embedded in the metadevices exhibits hysteretic behaviour with respect to external gate voltage^26^ that, in turn, leads to the hysteretic response in the effective properties of metadevices. Hence, their amplitude, phase and polarization states of light could be stored to multi-states with an appropriate MA structure. All stored states were stably maintained over 10^5^ s even in the absence of additional electrical stimuli, from which a retention time over 10 years can be anticipated at room temperature. Beyond the single electrical input memory function, the operational principle has been proven to be scalable to a multi-input system; for example, two-input logic-gate operations such as AND, OR and XOR (complementary NOR, NAND and XNOR, respectively) were implemented. Furthermore, it is shown that the same logic-gate metadevice can be reconfigured to operate as a two-bit digital-to-analogue convertor by a slight change in the operating condition.

## Results

### Graphene–ferroelectric nonvolatile memory metadevice

**Figure 1:**
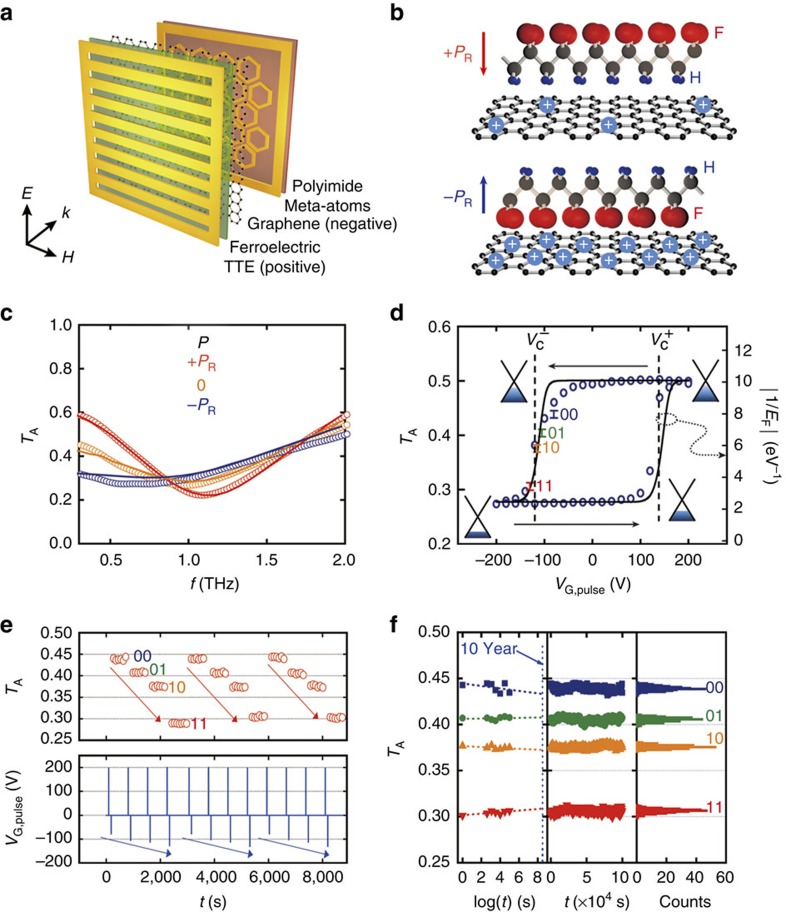
Graphene–ferroelectric amplitude and phase memory metadevice. (**a**) Schematic representation of the graphene–ferroelectric memory metadevice composed of a THz transparent electrode (TTE) with periodical metallic lines (4 μm linewidth and 2 μm spaces between the lines), ferroelectric polymer layer (2.1 μm, represented by green), single-layer graphene, hexagonal MAs and polyimide (1 μm, represented by light red) as substrate. Polarization of the incident THz is perpendicular to the TTE lines. (**b**) Schematic representation of the principle of nonvolatile doping in inherently p-doped graphene by ferroelectric polarization (*P*). Positive external voltage induces +*P*_R_ at the surface of a ferroelectric, resulting in hole depletion in graphene while negative external voltage changes *P* to −*P*_R_, thereby resulting in the accumulation of holes in graphene. (**c**) Measured (open circles) and simulated (solid lines) THz transmission spectra for external pulsed gating voltage (*V*_G,pulse_) lasting for 1 s. The red, blue and yellow lines and circles represent the results of application of *V*_G,pulse_(+200 V), *V*_G,pulse_(−200 V) and *V*_G,pulse_(−120 V), respectively. (**d**) Hysteresis in the measured transmission amplitude (*T*_A_) and the calculated Fermi level of graphene for *V*_G,pulse_ within a range of +200 and −200 V at a specific frequency of 0.5 THz. 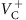 and 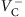 are the positive and negative coercive voltages, respectively. Arrow refers to the *V*_G,pulse_ sweep direction. Logic states denoted as 00, 01, 10 and 11 correspond to the multi-level transmission amplitudes for the retention time measurement. Error bars indicate the variation of each measured logic state. (**e**) Timing diagram of the transmission amplitude (*T*_A_) measured at 0.5 THz for various *V*_G,pulse_ values of −80, −105, −115 and −130 V. Counting from the highest transmission amplitude, data states were designated as 00, 01, 10 and 11. Before each *V*_G,pulse_ was applied, *V*_G,pulse_(+200 V) was applied to reset the ferroelectric polarization to +*P*_R_. (**f**) Transmission amplitude (*T*_A_) retention time measured at 0.5 THz for 1 × 10^5^ s and a histogram for each state.

A schematic representation of the graphene–ferroelectric nonvolatile memory metadevice (GF-NMM) is depicted in Fig. 1a. An array of hexagonal MAs, single-layer graphene, a ferroelectric polymer (poly(vinylidene fluoride-*co*-trifluoroethylene) or P(VDF-TrFE)) and a terahertz transparent electrode (TTE) composed of periodical subwavelength-scale metallic strips are placed sequentially on a polyimide substrate (fabrication details are described in the Methods, Supplementary Fig. 1 and Supplementary Note 1). An array of the hexagonal metallic pattern exhibiting polarization-independent inductance–capacitance (*LC*) resonance was chosen as the MA structure to intensify light–matter interaction. A large-size single-layer graphene synthesized by chemical vapour deposition^27^ on a Cu foil is transferred onto the array of MAs on polyimide using a ferroelectric polymer as a mechanically supporting film^28^. Since the dipoles in the ferroelectric polymer, P(VDF-TrFE), consist of weakly electronegative hydrogen atoms and strongly electronegative fluorine atoms, the application of an external gate voltage (*V*_G_) over the coercive voltage (*V*_C_) aligns the dipoles in the ferroelectric. These aligned dipoles, as a result, induce polarization (*P*) at the surface of the ferroelectric and exert an electrostatic force consistently on the charge carriers in the graphene layer (Fig. 1b). The TTE was carefully designed to apply a uniform electric field to the ferroelectric and transmit broadband terahertz waves vertically incident on the TTE without much loss as in previous works^29^. Pulsed external gate voltage (*V*_G,pulse_(*V*)) lasting for 1 s was applied between the TTE and the graphene/MAs in the measurement.

Terahertz time domain spectroscopy (THz-TDS) was carried out to characterize the fabricated GF-NMM as shown in Fig. 1c. On applying *V*_G,pulse_(+200 V) *P,* corresponding to positive remanent polarization (+*P*_R_), depletes the same polar charges out of graphene; THz transmission spectra through the GF-NMM showed a resonance dip at 1.1 THz. With the subsequent application of *V*_G,pulse_(−200 V), *P* changes to negative remanent polarization (−*P*_R_); the resonance frequency then shifted to 0.8 THz and the bandwidth was observed to slightly broaden.

Figure 1d shows the measured transmission amplitude (*T*_A_) through the GF-NMM and the quantitatively estimated Fermi level (*E*_F_) of graphene in the GF-NMM as a function of *V*_G,pulse_. Experimentally observed hysteretic variation of the spectral features is attributed to the change in graphene doping level resulting from the reversal of ferroelectric *P* (Supplementary Figs 2,3 and Supplementary Discussion). It is worthwhile to note that gradual transmission change is observed near positive and negative *V*_C_ and multi-state memory operation can be achieved by utilizing this gradual change near *V*_C_. To validate multi-state memory operation, a timing diagram of *T*_A_ was recorded at 0.5 THz for various *V*_G,pulse_ values of −80, −105, −115 and −130 V and plotted in Fig. 1e. From the highest *T*_A_, data states were designated as 00, 01, 10 and 11. Before addressing each state, *V*_G,pulse_(+200 V) was applied to reset ferroelectric polarization to +*P*_R_.

Reliable nonvolatile memory operation requires not only data separation but also long retention time. Figure 1f depicts the retention time for each stored *T*_A_ state; the GF-NMM has been observed to retain state information for all the states for over 10^5^ s (the standard variation for *T*_A_, *σ*_state_ is in the range between 5.62 × 10^−3^ and 7.58 × 10^−3^). The extrapolated *T*_A_ for all states do not cross each other for over 8.5 × 10^8^ s (∼10 years), which confirms that the demonstrated GF-NMM has indisputable nonvolatility. An additional measurement on THz-TDS system stability revealed that the slight variation in the *T*_A_ was mostly a result of the fluctuations of the femtosecond laser output that was used for the THz-wave generation (the fluctuation of our femtosecond laser, *σ*_laser_, was 4.43 × 10^−3^; Supplementary Fig. 4 and Supplementary Table 1). As the MA structure used in the measurement is of a resonant type, the phase tunability and nonvolatility were also measured. In the case of phase response, *V*_G,pulse_-dependent hysteretic behaviour and multi-state stable retention properties were found to be similar to the transmission amplitude response (Supplementary Fig. 5).

Doping concentration in graphene is more resistant to change with time when graphene–ferroelectric hybrid devices operate in the accumulation mode compared with the depletion mode, especially at the beginning of the retention measurement^30^. Because the graphene used in our experiments is inherently p-doped and the intrinsic doping concentration (6.17 × 10^12^ cm^−2^) is larger than the extrinsic doping concentration modulated by the ferroelectric polarization (6.08 × 10^12^ cm^−2^), our device operates exclusively in the p-doped regime (Supplementary Fig. 6 and Supplementary Note 2). Moreover, all multi-states were addressed by applying a negative *V*_G,pulse_ for further accumulation mode, so that the time-dependent drift in the *E*_F_ of graphene in the GF-NMM is negligible, and, therefore, stable operation is possible. Ferroelectric polarization switching depends on the magnitude and duration of *V*_G,pulse_. For an electric field of 1 MV cm^−1^ corresponding to *V*_G,pulse_(+200 V), previous studies have reported a polarization switching time of ∼1 ms (refs 31, 32). Approximately 10 times faster switching would be expected by a *V*_G,pulse_ with a one and a half times greater amplitude^32^.

**Figure 2:**
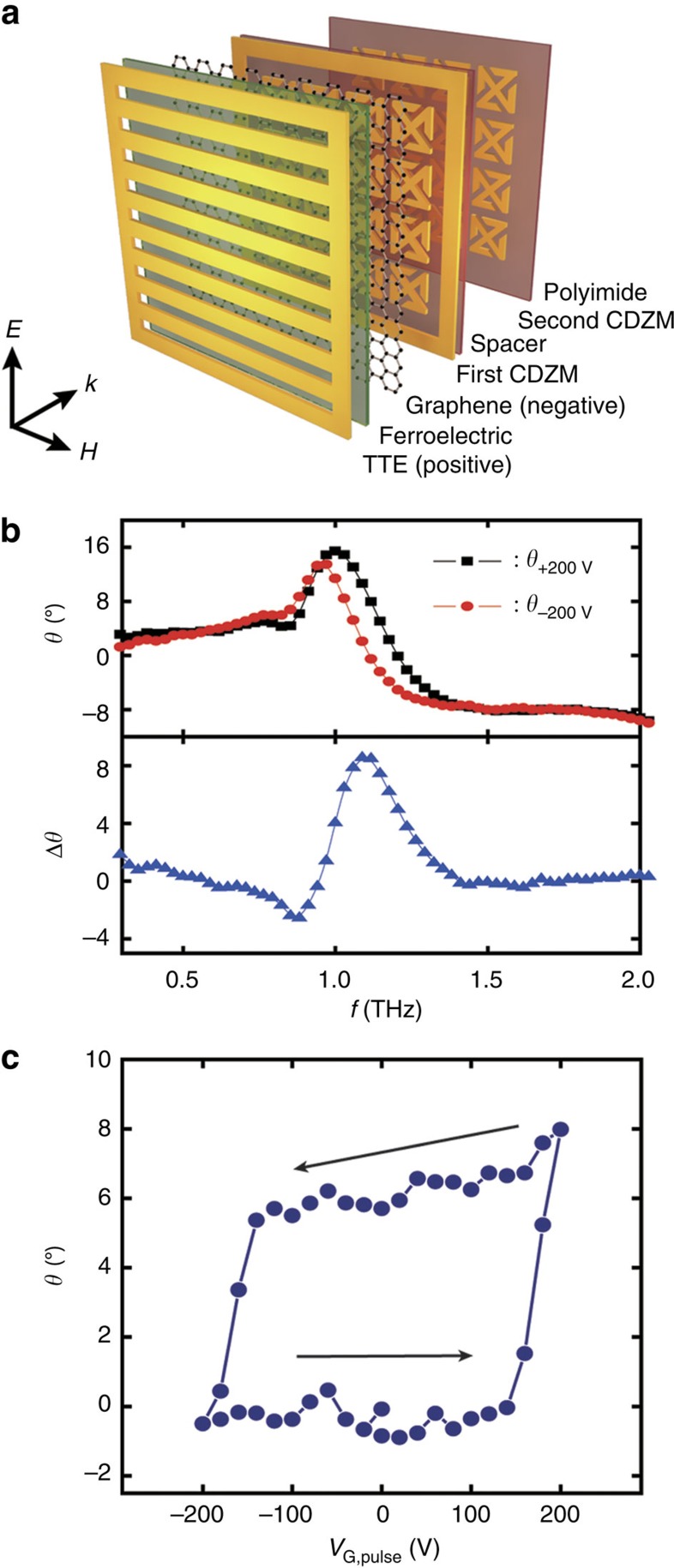
Graphene–ferroelectric chiral memory metadevice. (**a**) Schematic representation of the graphene–ferroelectric chiral memory metadevice composed of THz transparent electrode (TTE), ferroelectric polymer layer (2.1 μm, represented by green), single-layer graphene, CDZM with a spacer of 2 μm and a polyimide (1 μm, represented by light red) as the substrate. Polarization of the incident THz is perpendicular to the TTE lines. (**b**) Polarization rotation angle (*θ*) through chiral memory metadevice after the application of an external pulsed gating voltage (*V*_G,pulse_) lasting for 1 s, and the difference (Δ*θ*) between *θ*_+200V_ and *θ*_−200V_. (**c**) Polarization rotation angle (*θ*) in the *V*_G,pulse_ within a range of +200 and −200 V at 1.1 THz. Arrows refer to the *V*_G,pulse_ sweep direction. CDZM, conjugated double Z meta-molecules.

The principle of amplitude and phase memory operation can also be applied to light polarization memory with a chiral metamaterial (chiral GF-NMM), in which the plane of linearly polarized incident light is rotated as the light travels through. Recently, it was shown that with the integration of active materials into metamaterials, polarization switching and modulation can be dynamically controlled^33^. However, optical activity such as circular dichroism and polarization rotation in most metamaterials can so far only be tuned by continuous external optical stimuli. With the incorporation of graphene and a ferroelectric into the chiral metamaterials, the polarization states of light passing through a chiral GF-NMM can be stored by *V*_G,pulse_(V). Strongly coupled chiral meta-molecules (MM) (ref. 34) were employed in the fabrication of the chiral GF-NMM as shown in Fig. 2a. The rest of the chiral GF-NMM is identical with the amplitude and phase GF-NMM described above (Supplementary Fig. 7 and Supplementary Note 3).

Figure 2b shows the azimuthal polarization rotation angle (*θ*) for two distinct *V*_G,pulse_ values. Here, *θ* is extracted from the phase difference between the two circular polarizations (see Methods). On applying *V*_G,pulse_(+200 V), *θ*_+200V_ shows a maximum value of 15° at 1.0 THz, while *θ*_−200V_ exhibits a maximum value of 14° at 0.9 THz. It can be seen from Fig. 2b that *Δθ* (=*θ*_+200V_ to *θ*_−200V_) attains the maximum value of 8° at 1.1 THz. To trace the hysteretic behaviour in the polarization states more clearly, *θ* measurement was carried out at 1.1 THz and is plotted in Fig. 2c. Because of ferroelectricity, *θ* changes gradually near positive and negative *V*_C_. Multi-level polarization states can also be stored for over 10^5^ s without much degradation as shown in the operation of three different polarization states (Supplementary Fig. 8).

### Graphene–ferroelectric reconfigurable logic-gate metadevice

**Figure 3:**
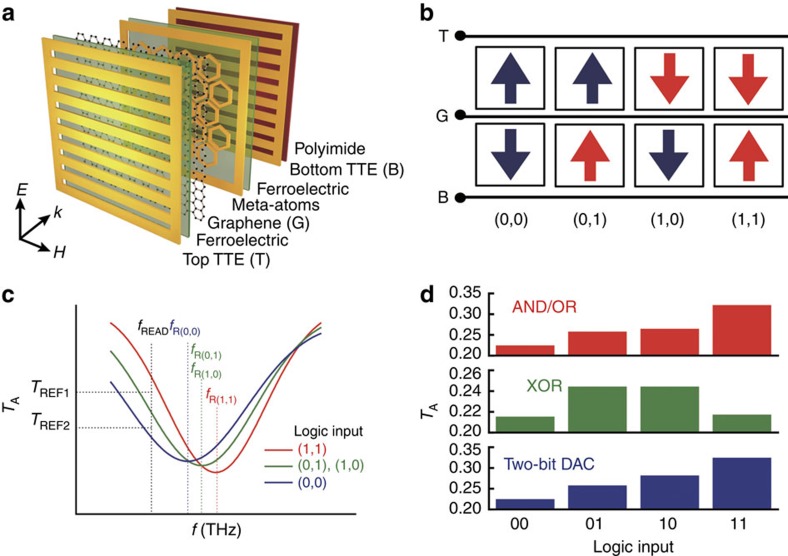
Graphene–ferroelectric reconfigurable logic-gate metadevice. (**a**) Schematic representation of the graphene–ferroelectric reconfigurable logic-gate metadevice composed of a top THz transparent electrode (top; TTE, T), a ferroelectric polymer layer (2.1 μm, represented by green), a single-layer graphene, hexagonal MAs, a ferroelectric polymer layer (2.1 μm, represented by green), a bottom THz transparent electrode (bottom; TTE, B) and a polyimide layer (1 μm, represented by light red) as the substrate. Polarization of the incident THz is perpendicular to the two TTE lines. (**b**) Schematic representation of the four kinds of polarization alignments for input logic states. Red arrow implies an application of positive pulsed gating voltage for logic state 1 and blue arrow refers to the application of negative pulsed gating voltage for logic state 0. (**c**) Schematic representation of the transmission spectra for input logic states. For each input logic state, the relationship *f*_R(0,0)_<*f*_R(0,1)_=*f*_R(1,0)_<*f*_R(1,1)_ is satisfied in the graphene–ferroelectric reconfigurable logic-gate metadevice because of p-doped graphene. A frequency of *f*_READ_ was designated for data reading and two reference transmission amplitudes, *T*_REF1_ and *T*_REF2_ were defined to execute AND (complementary NOR) and OR (complementary NAND) gate operations. For AND gate operation, the reference transmission amplitude is set to *T*_REF1_. For OR gate operation, the reference is *T*_REF2_. (**d**) Experimental transmission amplitude (*T*_A_) measured at 0.5 THz for the four types of logic inputs in the AND/OR gates, the XOR gate and the two-bit DAC.

The underlying concept for the operation of GF-NMM can be extended to a multi-input system such as a reconfigurable logic-gates metadevice (graphene–ferroelectric reconfigurable logic-gate metadevice (GF-RLM)). For example, a two-input system can be implemented by encapsulating graphene within two controllable ferroelectric layers as shown in Fig. 3a (Supplementary Fig. 9 and Supplementary Note 4). Independent pulsed gate control (*V*_G,pulse_) of each ferroelectric layer and the resulting combination of polarizations offered by the individual ferroelectric layers can lead to an increase in the degree of freedom in the manipulation of carrier concentration in graphene (*N*_G_) when compared with the GF-NMM having a single ferroelectric layer. Corresponding to the combination of two electrical inputs (the top electrode (T) and the bottom electrode (B)), *N*_G_ as well as the THz transmission through the two-input system is expected to give unique logic outputs. Figure 3b shows the combination of polarization values in the ferroelectric layers that correspond to four kinds of logic inputs, (0, 0), (0, 1), (1, 0) and (1, 1). Input logic states 1 and 0 are prepared by applying *V*_G,pulse_(+200 V) and *V*_G,pulse_(−200 V) to the corresponding ferroelectric layers, respectively. If graphene in GF-RLM is inherently p-doped, logic input (1, 1) depletes majority carriers (holes) and logic input (0, 0) accumulates holes in the graphene. In the two intermediate states, (0, 1) and (1, 0), the hole concentration in graphene will be set to the values that are between those corresponding to input states (0, 0) and (1, 1). The variation in *N*_G_ results in a change in the transmission spectrum as shown schematically in Fig. 3c, in which the resonance frequency is red-shifted as *N*_G_ increases^29^. With the appropriate frequency choice for data reading (*f*_READ_), the logic output can be decoded by comparing with *T*_REF1_ for the AND (complementary NOR) operation or *T*_REF2_ for the OR (complementary NAND) operation. Furthermore, XOR (complementary XNOR) operation can also be realized by simply rearranging the electric connections Supplementary Figs 10,11). All the logic-gate operations are measured using THz-TDS and shown in Fig. 3d (Supplementary Fig. 12 and Supplementary Table 2).

In addition to the logic operations, the device can also be configured as a digital-to-analogue converter if the remanant polarization in each of the ferroelectric layers assumes a different value. If the two ferroelectric layers supply two different *P*_R_ values, four kinds of *N*_G_ levels corresponding to (+*P*_R1_, +*P*_R2_), (+*P*_R1_, −*P*_R2_), (−*P*_R1_, +*P*_R2_) and (−*P*_R1_, −*P*_R2_) are possible, which implies that *f*_R(0,1)_ is different from *f*_R(1,0)_ as shown in Fig. 3c. The effective method to control polarization switching was demonstrated in a prior work^35^, in which ferroelectric switching was controlled by setting the current limitation (*I*^C^, compliance current). By setting different compliance current for bottom and top TTE, four distinct levels of transmission amplitude were measured as shown in Fig. 3d (Supplementary Fig. 12). The combination of two digital inputs resulting in four levels of optical analogue states validates the two-bit digital-to-analogue convertor operation. Although the transmission loss and optoelectric conversion should be considered in the compact integration of diverse functional metadevices, the platform and the principle of operation provided here might be extended to a certain class of multi-input systems in principle.

## Discussion

In this work, electrically programmable nonvolatile memory and reconfigurable logic-gate metadevices were demonstrated with the hybridization of graphene, a ferroelectric and MAs/molecules. These functional metadevices are the first demonstration of superior nonvolatility and logic-gate operation at room temperature, which offer new pathways for emerging optoelectronic applications. Nonvolatile memory function liberates active metadevices from immovable power supply units, leading to saving of energy resources. Reconfigurability presents a user-oriented general-purpose metadevice that can be configured through electrical programming. More complex functionality can be made possible by the compact integration of diverse functional metadevices and/or by the design changes of the demonstrated metadevices. Ultimately, high-end metasystems to perform advanced functionalities may be developed by grafting the concept of GF-NMM and GF-RLM onto the diverse architectures of graphene-based nonvolatile memory, as recently demonstrated in the field of electronic devices^36^.

## Methods

### Fabrication processes for the GF-NMM and GF-RLM

All metallic parts of the hexagonal MAs/molecules and the TTE were made of 100-nm thick Au with a 10 nm thick Cr layer for enhanced adhesive strength. Single-layer graphene was synthesized by chemical vapour deposition on a Cu foil (G/Cu). Poly(vinylidene fluoride-trifluoroethylene), P(VDF-TrFE), manufactured by MSI Sensors Inc. was chosen as the ferroelectric polymer.

For the nonvolatile memory metadevice, a polyimide (PI, PI-2610, HD MicroSystems) was spin-coated and cured on a Si wafer. An array of hexagonal MAs was deposited by a photolithography, thermal evaporation and lift-off process (MA/PI/Si). On a SiO_2_/Si wafer, a ferroelectric polymer (FP) was spin-coated, annealed at 130 °C for 1 h, and cooled down to room temperature slowly. A TTE was patterned by a photolithography, thermal evaporation of Cr/Au and lift-off process (TTE/FP/SiO_2_/Si). By etching the SiO_2_ with an HF aqueous solution, the TTE/FP was transferred onto graphene (G) on Cu foil. By etching the Cu foil with the Cu etchant APS-100, the TTE/FP/G was transferred onto the MA/PI/Si and thermally treated for adhesion. Finally, Si was detached mechanically. For the polarization state memory metadevice, MA/PI on Si was replaced with MM/PI, which was fabricated by stacking conjugated double Z patterns with a polyimide spacer of 2 μm.

For the reconfigurable logic-gate metadevice, an MA layer was deposited on G/Cu. An FP was spin-coated on the MA/G/Cu. In addition, a TTE as the top electrode was deposited on the FP/MA/G/Cu. By etching the Cu foil with a Cu etchant of APS-100, a TTE/FP/MA/G hybrid film was prepared. On a Si wafer sacrificial substrate, PI was spin-coated and TTE as the bottom electrode was deposited on PI/Si (TTE/PI/Si). An FP was spin-coated onto the TTE/PI/Si (FP/TTE/PI/Si). Reconfigurable logic-gate metadevice was fabricated by transferring the TTE/FP/MA/G onto the FP/TTE/PI/Si. Finally, Si was detached mechanically.

All metadevices were mounted on a punched printed circuit board for THz-TDS measurements.

### THz-TDS system

To generate the terahertz signal, we used a low-temperature grown GaAs THz emitter (Tera-SED, Gigaoptics) illuminated by a femtosecond Ti:sapphire laser pulse train of wavelength 800 nm and 80 MHz repetition rate, respectively. An electro-optic sampling method was used to detect the transmitted terahertz signals in the time domain by using a (110) oriented ZnTe crystal of 1 mm thickness. The THz-TDS system has a usable bandwidth of 0.3–2.5 THz and a signal to noise ratio (*S*/*N*) of over 10,000:1.

### Measurement of the chiral GF-NMM

The chiral GF-NMM was characterized using conventional THz-TDS. The metadevice was positioned between two wire-grid THz polarizers, that are mounted on motorized rotational stages with parallel or crossed configurations, to measure the co-polarized (*T*_||_) and cross-polarized (*T*_⊥_) transmission coefficients. The sample was carefully aligned to assure the TTE of the metadevice remains parallel to the front polarizer. From the measured transmission coefficients, the right and left circularly polarized transmission coefficients can be obtained as *T*_+_=*T*_||_+*iT*_⊥_ and *T*_−_=*T*_||_−*iT*_⊥_. The azimuthal rotation angle can be calculated by the phase retardation between two circularly polarized waves as 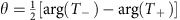.

## Additional information

**How to cite this article:** Kim, W. Y. *et al.* Graphene-ferroelectric metadevices for nonvolatile memory and reconfigurable logic-gate operations. *Nat. Commun.* 7:10429 doi: 10.1038/ncomms10429 (2016).

## Supplementary Material

Supplementary Information Supplementary Figures 1-12, Supplementary Tables 1-2, Supplementary Notes 1-4, Supplementary Discussion and Supplementary References
