# Supplementary material for: Graphene–ferroelectric metadevices for nonvolatile memory and reconfigurable logic-gate operations
Source: Nat Commun. 2016 Jan 27;7:10429. doi: 10.1038/ncomms10429 (PMC4737808; doi:10.1038/ncomms10429)
Supplement: Supplementary Information — Supplementary Figures 1-12, Supplementary Tables 1-2, Supplementary Notes 1-4, Supplementary Discussion and Supplementary References [file ncomms10429-s1.pdf]

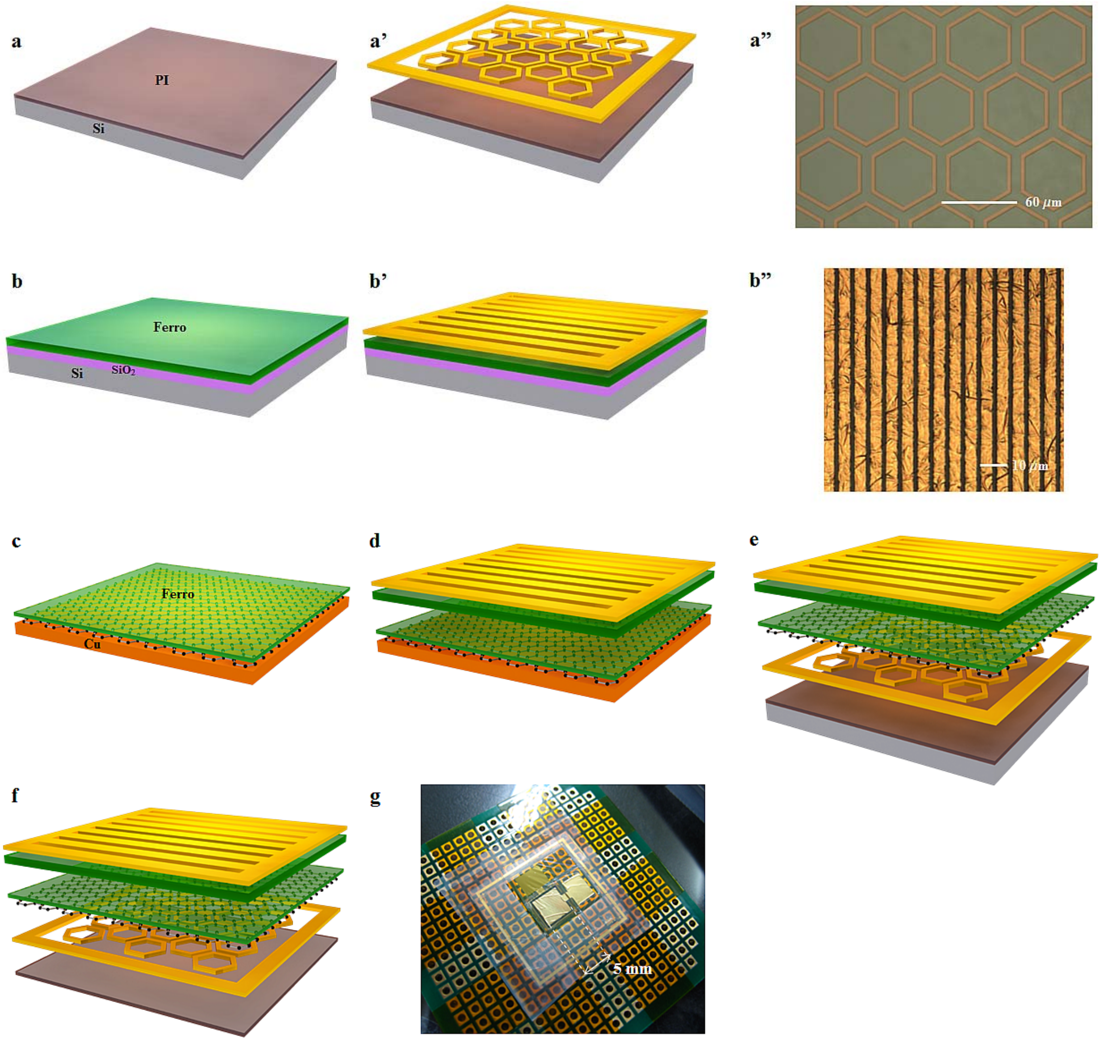

Supplementary Figure 1. Fabrication process of GF-NMM and its optical images (a) Polyimide deposition on a bare Si wafer **(a')** Formation of a hexagonal meta-atom array on PI/Si **(a")** Optical microscopy image of the hexagonal meta-atom array where the scale bar is 60 μm, the gap between adjacent hexagons is 8 μm, and the metal line width is 3 μm. **(b)** Ferroelectric film deposition on SiO_2/Si **(b')** Formation of the terahertz transparent electrode on the Ferro/SiO_2/Si **(b")** Optical microscopy image of terahertz transparent electrodes where the scale bar is 10 μm, the gap between adjacent metal lines is 2 μm, and the metal line width is 4 μm. **(c)** Thin ferroelectric film deposition on the graphene-grown Cu foil **(d)** Transferring TTE/Ferro onto Ferro/G/Cu **(e)** Cu-etching and transferring of the TTE/Ferro/G onto MA/PI/Si **(f)** Removal of Si by mechanical detaching and **(g)** mounting of the

TTE/Ferro/G/MA/PI onto punched printed circuit board (PCB). Scale bar is 5 mm. Detailed conditions are described in Supplementary Note 1.

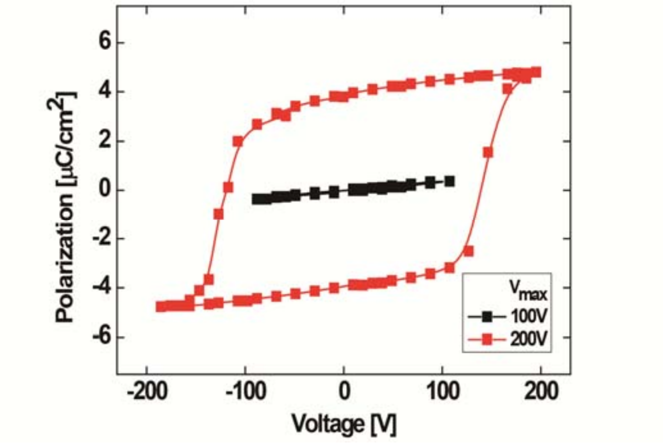

Supplementary Figure 2. Polarization-voltage hysteresis of the TTE / P(VDF-TrFE) / graphene capacitor The measurement at V_{MAX} = 200 V showed a remanent polarization (P_R) of 4.1 μC/cm^2 and a coercive voltage (V_C) of 129 V. Thus a coercive field (E_C) of 0.61 MV/cm could be deduced. Moreover, a dielectric constant (ε_{Ferro}) of P(VDF-TrFE) can be estimated to be 8.78 from the slope measurement (dP/dV) at V_{MAX} = 100 V. Therefore, the doping coefficient α of graphene is found to be 2.3 × 10^{10} $cm^{-2}V^{-1}$ for the 2.1 μm-thick P(VDF-TrFE) layer.

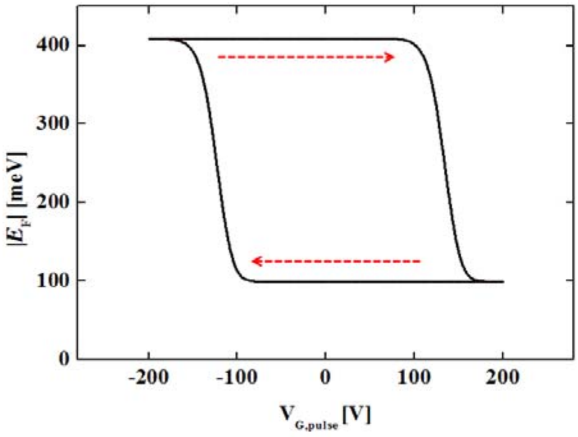

Supplementary Figure 3. Fermi level of graphene in memory metadevice calculated with parameters extracted from the electrical measurements and FDTD simulation

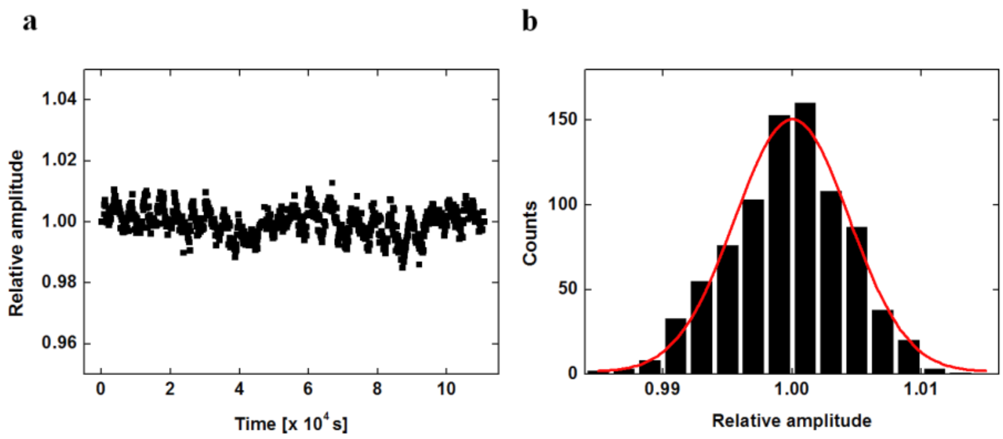

Supplementary Figure 4. THz-TDS system stability measurements (a) relative amplitude for 1.1×10^5 s **(b)** histogram of measured data and its fitted Gaussian curve.

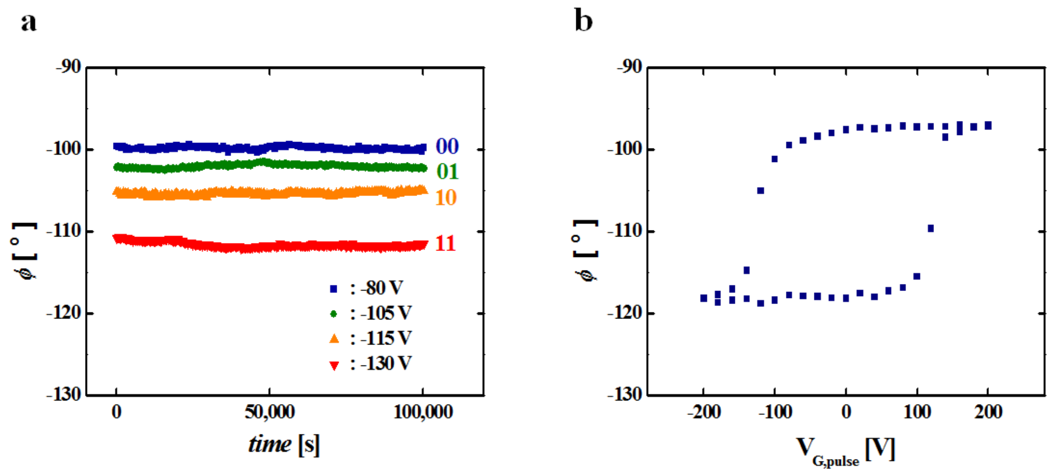

Supplementary Figure 5. Phase response at 0.5 THz (**a**) Retention time of phase responses induced by various $V_{G,pulse}$ values. Logic states of 00, 01, 10, and 11 are designated by application of $V_{G,pulse}$ of -80, -105, -115 and -130 V, respectively. Each logic state was addressed after the application of a reset $V_{G,pulse}$(+200 V) for initializing polarization to $+P_R$. (**b**) Phase response of GF-NMM measured at 0.5 THz

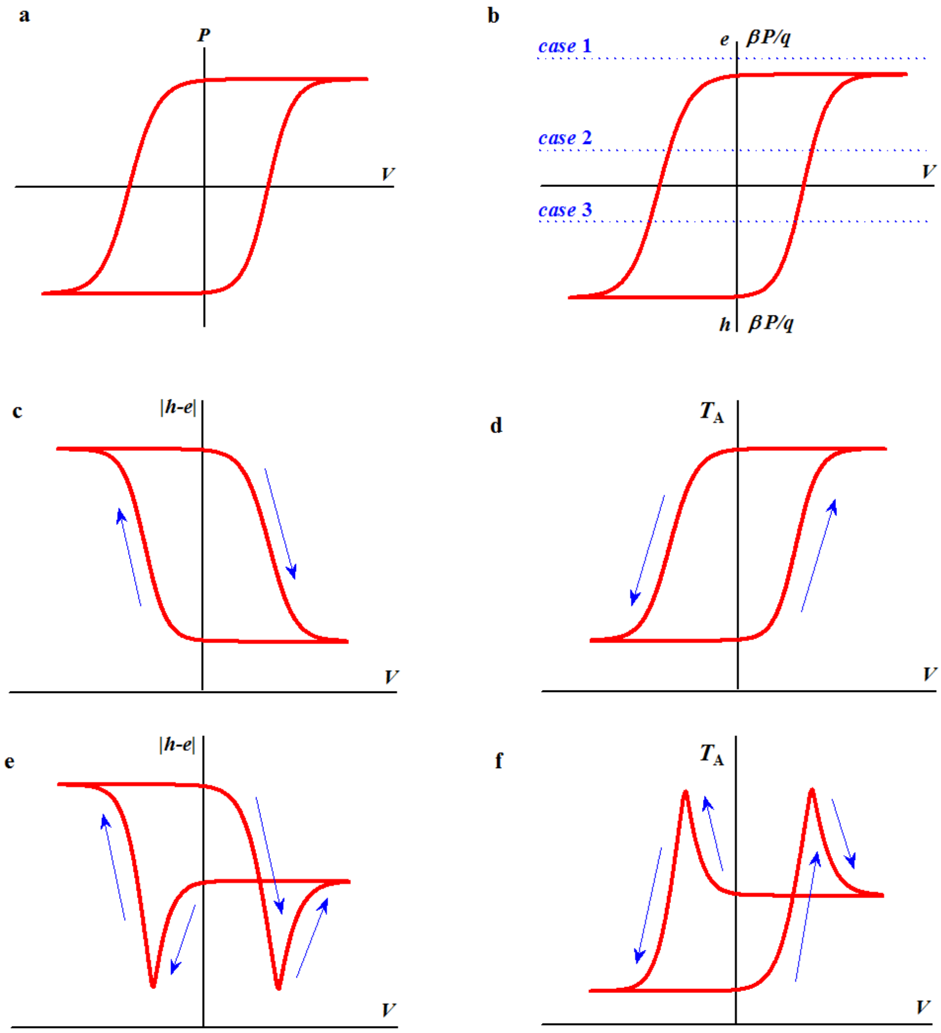

Supplementary Figure 6. The gating voltage dependence of graphene doping concentration and transmission amplitude T_A at off-resonance frequency in GF-NMM shown schematically (**a**) polarization-voltage relationship in a ferroelectric material (**b**) carrier concentration induced in graphene, positive region *e* refers to electron and negative region *h* refers to hole on the y-axis. Case 1 corresponding to $\alpha V_{CNP} > \beta P_R/q > 0$, case 2 to $\beta P_R/q > \alpha V_{CNP} > 0$, and case 3 to $\alpha V_{CNP} < 0$. (**c**) Net carrier concentration for the case 1. (**d**) Transmission amplitude (T_A) in case 1 at an off-resonant frequency. (**e**) Net carrier concentration in the case 2. (**f**) Transmission amplitude (T_A) in the case 2 at an off-resonant frequency. Arrows in (c)-(f) refer to the voltage sweep direction. Detailed explanation is described in Supplementary Note 2.

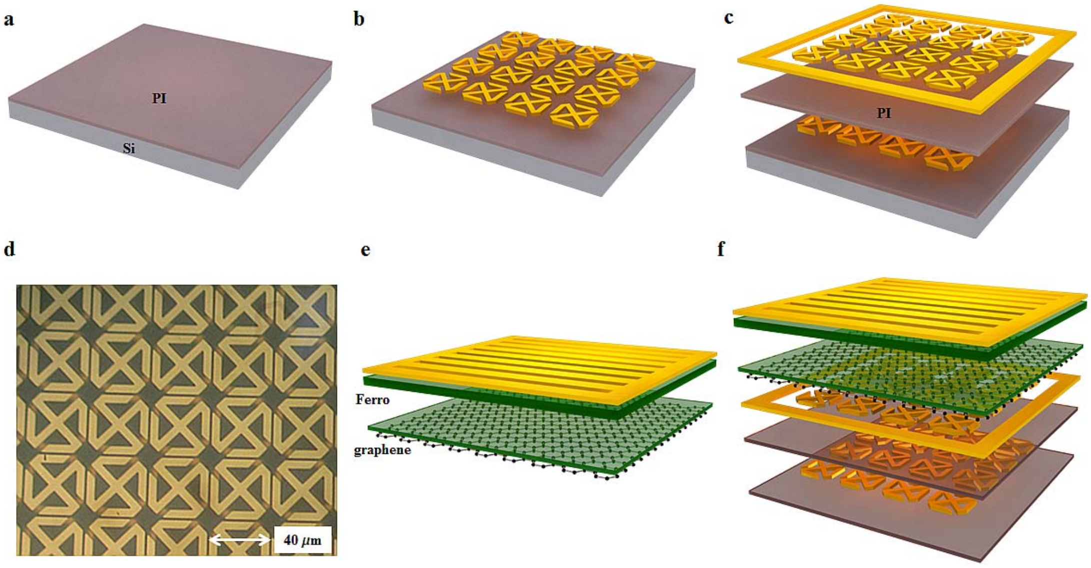

Supplementary Figure 7. Fabrication process of chiral GF-NMM and its optical image (a) Polyimide deposition on bare Si wafer **(b)** Formation of conjugated double Z meta-molecule array on PI/Si **(c)** Deposition of polyimide spacer and the formation of second conjugated double Z meta-molecule array **(d)** Optical microscopy image of MM: scale bar is 40 μm, gap between adjacent CDZs is 1.5 μm, and the metal line width is 4 μm. **(e)** Preparation of TTE/Ferro/G hybrid film **(f)** Transferring TTE/Ferro/G onto MM/PI/Si and Si removal by mechanical detachment. Detailed conditions are described in Supplementary Note 3.

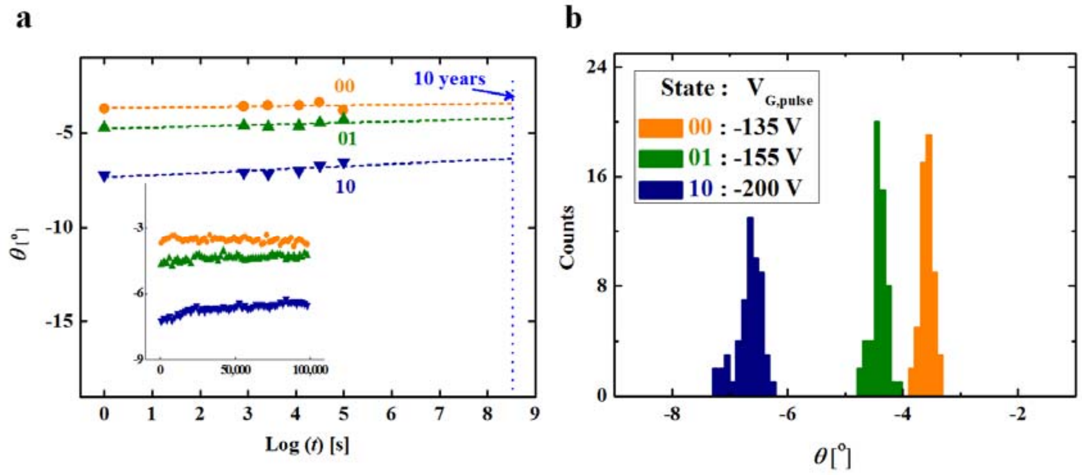

Supplementary Figure 8. Retention time measurement for the rotation angle of chiral GF-NMM (**a**) Retention performance of rotation angle for three different states 00, 01, and

10. (**b**) Histogram for the retention data. A bias of -8° could be observed in the measured rotation angle (θ). However, this biased angle was not an intrinsic phenomenon but an offset that originated from the THz-TDS measurement system (polarization plate mismatch etc.).

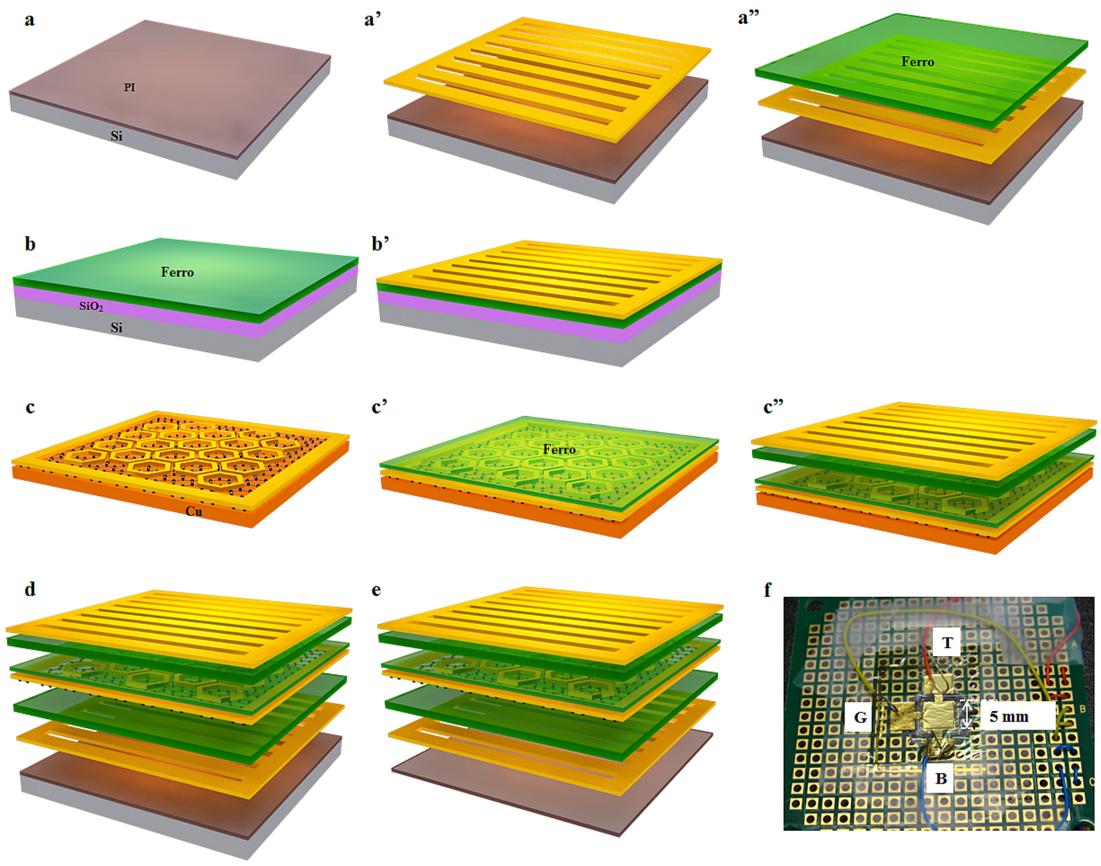

Supplementary Figure 9. Fabrication process of the GF-RLM and its optical image (a) Polyimide deposition on the bare Si wafer **(a')** Formation of the terahertz transparent electrode on PI/Si **(a")** Spin-coating of the ferroelectric film on TTE_2/PI/Si **(b)** Spin-coating of the ferroelectric film on SiO_2/Si **(b')** Formation of the terahertz transparent electrode on Ferro/SiO_2/Si **(c)** Formation of the meta-atom array on G/Cu **(c')** Spin-coating of a thin ferroelectric film on the graphene-grown Cu foil **(c")** Transfer of TTE_1/Ferro onto Ferro/MA/G/Cu **(d)** Cu etching and transfer of TTE_1/$Ferro_1$/MA/G onto $Ferro_2$/TTE_2/PI **(e)** Removal of Si and **(f)** mounting of TTE_1/$Ferro_1$/MA/G/$Ferro_2$/TTE_2/PI onto punched printed circuit board (PCB). Detailed conditions are described in Supplementary Note 4.

a

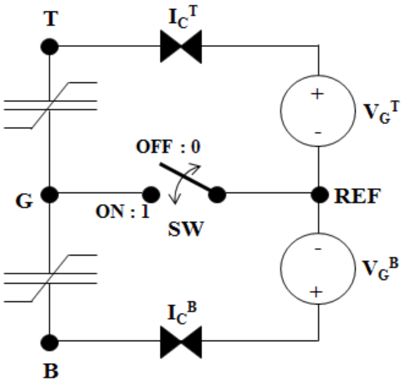

b

	SW	I_C^T [μA]	I_C^B [μA]
**AND/OR**	1	10	10
**XOR**	0	10	10
**2-bit DAC**	1	10	5

Supplementary Figure 10. Equivalent electrical circuit for the graphene-ferroelectric reconfigurable logic-gate metadevice and operational protocols. **(a)** I_C^T and I_C^B are the limiting currents of the functional parts of the top electrode (T) and the bottom electrode (B), respectively. When switch (SW) is ON:1, graphene (G) is connected to reference (REF), while SW:0 implies an open connection. **(b)** Operating conditions for the AND/OR gates, the XOR gate and the 2-bit digital-to-analogue convertor (DAC).

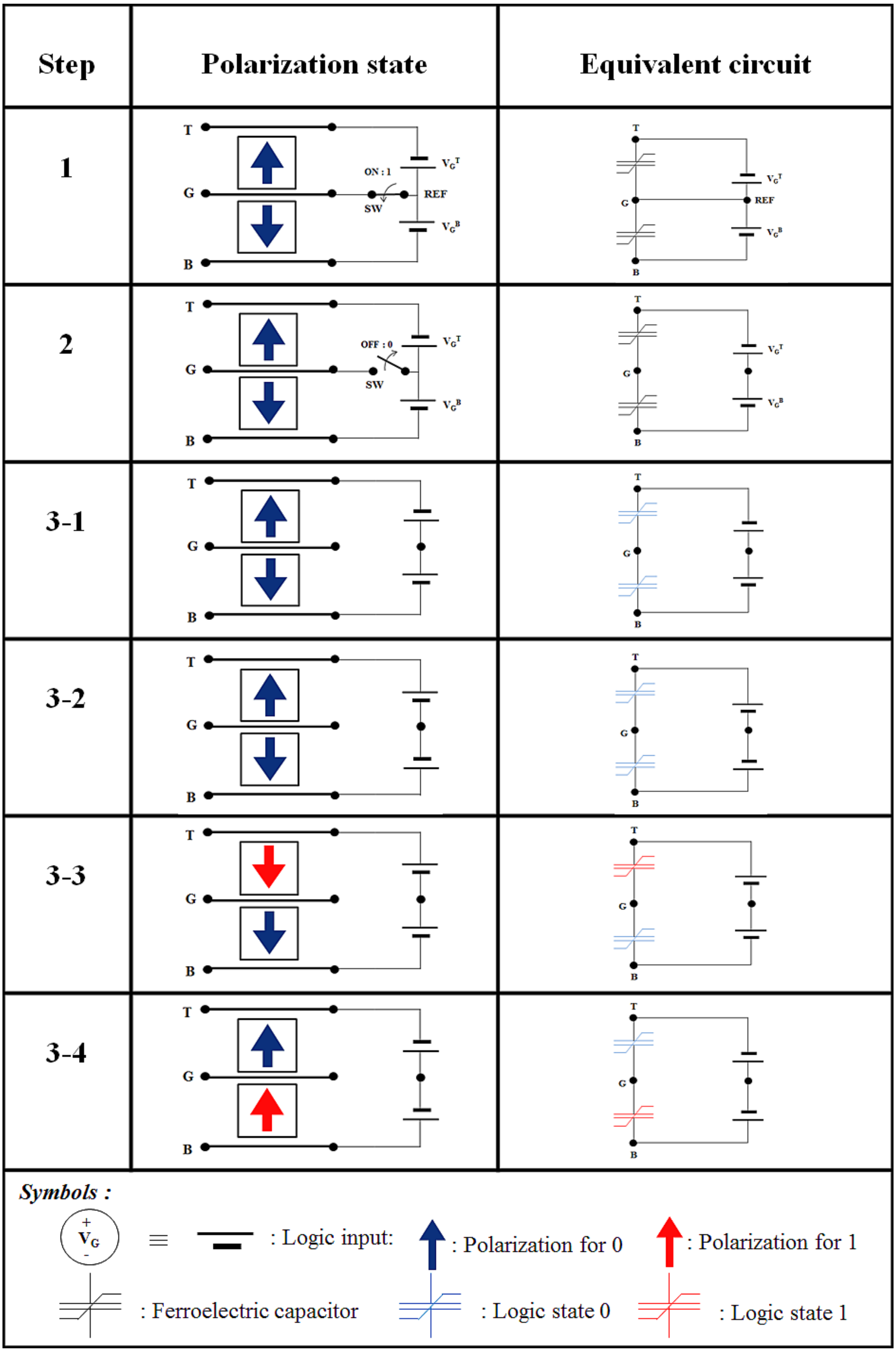

Supplementary Figure 11. Step-by-step operational schematics and the corresponding equivalent circuit for XOR gate operation (Step 1) Initialization to (0, 0) state, for which a

switch (SW) is closed (ON:1). **(Step 2)** The switch is opened (OFF:0) after initialization. **(Step 3)** XOR operations; Step 3-1 for (0, 0), Step 3-2 for (1, 1), Step 3-3 for (1, 0) and Step 3-4 for (0, 1). For (0, 1) and (1, 0) inputs, polarization switching in only one of two ferroelectric capacitors occurs so that the polarizations become parallel in both ferroelectric capacitors. As a result, the doping concentration in graphene is changed only for (0, 1) and (1, 0) inputs.

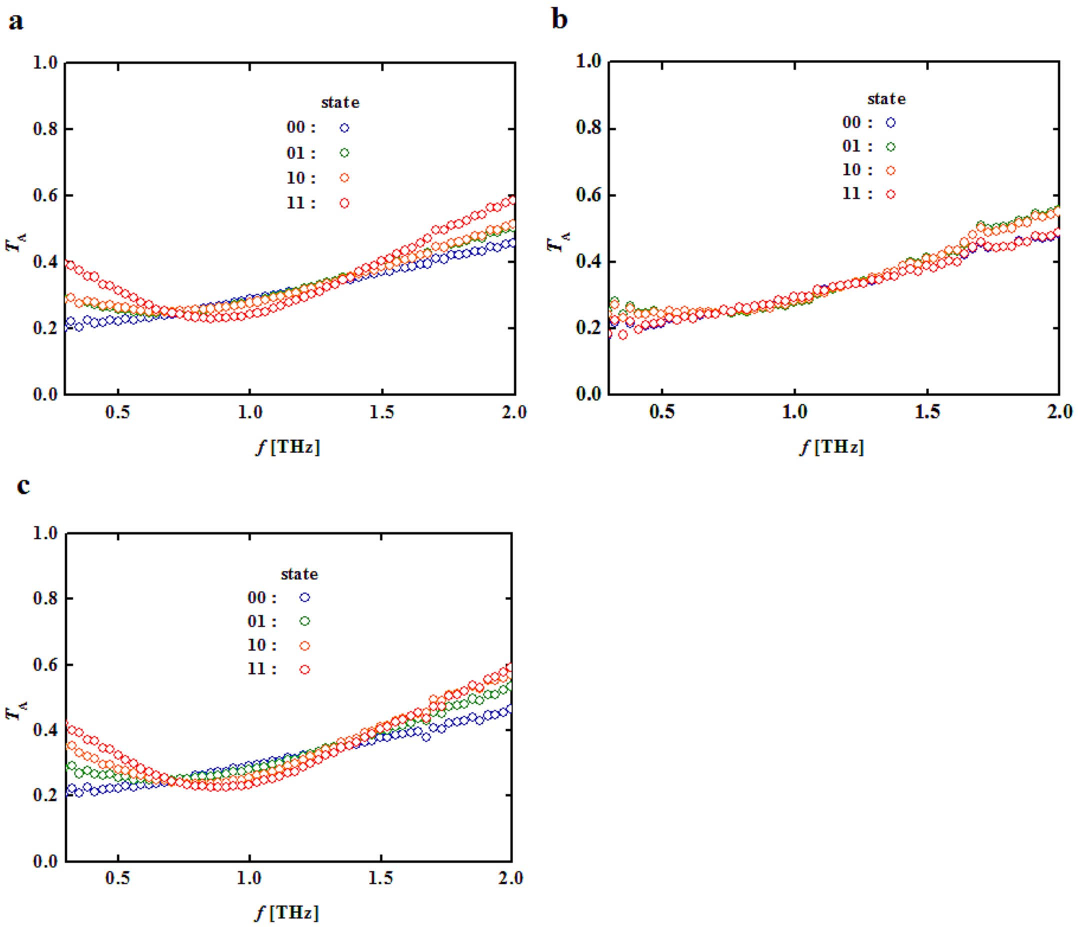

Supplementary Figure 12. Experimental THz-TDS spectra of logic-gates and 2-bit digital-to-analog convertor (DAC) operations (**a**) AND/OR (complementary NOR, NAND) gate operations. (**b**) XOR gate operation (**c**) 2-bit DAC operation. Experimental transmission amplitudes (T_A) at f_{READ} of 0.5 THz are summarized in Supplementary Table 2.

**Stored state**	**T_{AM} at 0.5 THz**	**σ**	**σ/T_{AM}**
**00**	**0.4391**	**2.54×10^{-3}**	**5.78×10^{-3}**
**01**	**0.4054**	**2.52×10^{-3}**	**6.22×10^{-3}**
**10**	**0.3756**	**2.11×10^{-3}**	**5.62×10^{-3}**
**11**	**0.3062**	**2.32×10^{-3}**	**7.58×10^{-3}**
**reference**	**1.0**	**4.43×10^{-3}**	**4.43×10^{-3}**

Supplementary Table 1. Statistics of transmission amplitude measured from THz-TDS, mean transmission amplitude (T_{AM}), standard deviation (σ) and normalized standard deviation (σ/T_{AM})

**V (T, B) / Function**	**(0.0)**	**(0,1)**	**(1,0)**	**(1,1)**
**AND / OR**	**0.224**	**0.257**	**0.264**	**0.321**
**XOR**	**0.215**	**0.244**	**0.244**	**0.217**
**2-bit DAC**	**0.224**	**0.257**	**0.281**	**0.324**

Supplementary Table 2. Experimental transmission amplitudes (T_A) at f_{READ} of 0.5 THz of logic-gates and 2-bit digital-to-analog convertor (DAC) operations

Supplementary Note 1:

On a silicon wafer, a commercialized polyimide (PI, product model: PI-2610, HD microsystems Inc.) layer was spin-coated (thickness = 1 μm) and cured (**PI/Si**). An array of hexagonal meta-atoms (MA) was formed on PI/Si using a negative photoresist (product model: AZ 2035, AZ Electronic Materials) and lift-off process (**MA/PI/Si**). On another SiO_2/Si wafer, a ferroelectric polymer film was spin-coated (thickness = 2 μm) and annealed at 130 °C for 1 h (**Ferro/SiO_2/Si**). For the preparation of ferroelectric polymer solution, poly(vinylidene fluoride-co-trifluoroethylene) (**Ferro**, P(VDF-TrFE), manufactured by MSI Sensors Inc.) was dissolved in cyclohexanone. The molar ratio of VDF:TrFE was 75:25. A terahertz transparent electrode (TTE, Cr/Au = 10 nm/100 nm) with a line width of 4 μm and a spacing of 2 μm was formed on Ferro/SiO_2/Si using a negative photoresist (product model: AZ 2035, AZ Electronic Materials) and lift-off process (**TTE/Ferro/SiO_2/Si**). To remove the photoresist residue from the lift-off process, a commercialized photoresist stripper (product model: AZ 400T, AZ Electronic Materials) was diluted with deionized water to 50 % [1]. Single layer graphene on Cu foil (**G/Cu**) synthesized by chemical vapour deposition was purchased from GRAPHENE SQUARE Inc. Due to rough surface morphology of G/Cu, a 100 nm thick P(VDF-TrFE) film was spin-coated on G/Cu (**Ferro/G/Cu**). The TTE/Ferro layer was detached by etching the SiO_2 substrate with hydrofluoric aqueous acid and transferred onto the Ferro/G/Cu without chemical and/or electrical degradation [1, 2]. To remove water and to reinforce the adhesive strength between TTE/Ferro and Ferro/G/Cu, the sample was thermally treated at 95 °C for 2 h (**TTE/Ferro/G/Cu**). The Cu foil was etched using APS-100 (ammonium persulfate aqueous solution, $(NH_4)_2S_2O_8$). The TTE/Ferro/G structure was transferred onto MA/PI/Si and the TTE/Ferro/G/MA/PI was separated from the Si substrate. The final sample was mounted on a punched printed circuit board (PCB) for THz-TDS measurements.

Supplementary Note 2:

Transmission amplitude (T_A) hysteretic behaviour depends on the relationship between the intrinsic doping concentration of graphene (αV_{CNP}) and the extrinsic doping concentration modulated by ferroelectric polarization ($\beta P/q$), where α is a doping coefficient, V_{CNP} is the charge neutral point, P is the ferroelectric polarization, β is a coupling constant described in Ref [3], and q is the elementary charge of 1.6×10^{-19} C.

In the case of $\alpha V_{CNP} > \beta P/q$ (*case 1* in Fig. 4b), the carriers in graphene are always holes as shown in Fig. 4c. Therefore, the doping concentration of graphene (N_G) increases monotonically as V_G changes from +200 V to -200 V, implying that the transmission amplitude T_A also changed monotonically.

However, in the case of $\alpha V_{CNP} < \beta P/q$ (*case 2* in Fig. 4b), the polarity of the carriers in graphene is changed from electron to hole as V_G moves from +200 V to -200 V, thereby implying that the transmission amplitude T_A hysteresis in Fig. 1d in the main manuscript must show a maximum T_A at a certain voltage position below +200 V.

The *case 3* in Fig. 4b indicates that the carriers in graphene are electrons, so transmission amplitude T_A behaviour will show counter-trend to the graphene in a p-type regime.

In conclusion, the graphene used for the GF-NMM is always in a p-type regime because the transmission amplitude T_A hysteresis behaviour in Fig 1d in the main manuscript indicates the trend described in Fig. 4d.

Supplementary Note 3:

On a silicon wafer, a commercialized polyimide (PI, product model: PI-2610, HD microsystems) layer was spin-coated (thickness = 1 μm) and cured (**PI/Si**). An array of conjugated double Z (CDZ) meta-molecule (MM) was formed on PI/Si using a negative photoresist (product model: AZ 2035, AZ Electronic Materials) and lift-off process (**MM/PI/Si**). The thickness of the polyimide spacer between two CDZs in a unit cell was 2 μm. The hybrid structure of terahertz transparent electrode (TTE, Cr/Au = 10 nm/100 nm), ferroelectric film (Ferro) and graphene (G) was fabricated in a manner similar to the process for the amplitude and phase GF-NMM, and was transferred onto the MM/PI/Si. After the

TTE/Ferro/G/MM/PI was detached from the Si substrate, the final sample was mounted on a punched printed circuit board (PCB) for THz-TDS measurements.

Supplementary Note 4:

On a silicon wafer, a commercialized polyimide (PI, product model: PI-2610, HD microsystems) layer was spin-coated (thickness = 1μm) and cured (**PI/Si**). A terahertz transparent electrode (TTE_2, Cr/Au = 10 nm/100 nm) was formed on PI/Si using a negative photoresist (product model: AZ 2035, AZ Electronic Materials) and lift-off process (**TTE_2/PI/Si**). A ferroelectric film of 2.1 μm was spin-coated on the TTE_2/PI/Si and annealed at 130 °C for 1 h (**$Ferro_2$/TTE_2/PI/Si**).

A single layer graphene on Cu foil (**G/Cu**) synthesized by chemical vapour deposition was purchased from GRAPHENE SQUARE Inc. On a G/Cu, an array of hexagonal meta-atoms (MA) was formed using a negative photoresist (product model: AZ 2035, AZ Electronic Materials) and lift-off process (**MA/G/Cu**). For planarization of the MA/G/Cu surface, a 100 nm thick ferroelectric film was spin-coated on it.

On another SiO_2/Si wafer, a ferroelectric polymer film was spin-coated (thickness = 2 μm) and annealed at 130 °C (**Ferro/SiO_2/Si**). A terahertz transparent electrode (TTE_1, Cr/Au = 10 nm/100 nm) was formed on the Ferro/SiO_2/Si using a negative photoresist (product model: AZ 2035, AZ Electronic Materials) and lift-off process (**TTE_1/Ferro/SiO_2/Si**). To remove the photoresist residue for the lift-off process, a commercialized photoresist stripper (product model: AZ 400T, AZ Electronic Materials) was diluted with deionized water to 50% [1].

The TTE_1/Ferro was detached by etching the SiO_2 substrate and transferred onto the Ferro/MA/G/Cu. To remove water and reinforce adhesion between the TTE_1/Ferro and the Ferro/MA/G/Cu, the sample was thermally treated at 95 °C for 2 h. The Cu foil was etched by APS-100 (ammonium persulfate aqueous solution, $(NH_4)_2S_2O_8$). Thus **TTE_1/$Ferro_1$/MA/G** was prepared.

Finally, the TTE_1/$Ferro_1$/MA/G was transferred onto the $Ferro_2$/TTE_2/PI/Si and the **TTE_1/$Ferro_1$/MA/G/$Ferro_2$/TTE_2/PI** was separated from the Si substrate. The final sample was mounted on a punched printed circuit board (PCB) to measure the THz-TDS. In the main manuscript, TTE_1 and TTE_2 are designated as top TTE (T) and bottom TTE (B), respectively.

Supplementary Discussion:

Using the optical dielectric constants (refractive index) of the polyimide and the ferroelectric films, the Fermi level (E_F) and the scattering time (τ) of graphene were extracted from the THz-TDS measurement. The Fermi level is a function of the total charge carrier concentration in graphene (N_G) and can be written as

$$E_F = \hbar \nu_F \sqrt{\pi N_G} \ , \tag{1}$$

where $\hbar$ is the Plank constant divided by 2π, and $\nu_F \approx 1\times10^6$ m/s is the Fermi velocity. The total charge carrier concentration N_G consists of the residual carrier concentration (n_{res}), the gating field-induced carrier concentration ($\alpha|V_G\text{-}V_{CNP}|$) and the ferroelectric polarization-induced carrier concentration of $\beta P/q$ (P is the ferroelectric polarization, β is a coupling constant described in Ref [3], and q is the elementary charge of 1.6×10^{-19} C).

$$N_G = \sqrt{(n_{res}^2 + n^2)} \ , \tag{2}$$

$$n = \alpha|V_G - V_{CNP}| \pm \beta\left(\frac{P}{q}\right) \ , \tag{3}$$

Therefore, E_F can be expressed as follows.

$$E_F = \hbar \nu_F \sqrt{\pi}\left(n_{res}^2 + \left(\alpha|V_G - V_{CNP}| \pm \beta\left(\frac{P}{q}\right)\right)^2\right)^{1/4} , \tag{4}$$

Here, $\alpha = 2.3 \times 10^{10}$ $cm^{-2}V^{-1}$ is the doping coefficient of the ferroelectric film of 2.1 μm thickness in Fig. 11 and V_{CNP} is the charge neutral point of graphene.

Because of nonvolatility, the term V_G can be removed from the expression for the steady state after the application of an external gating pulse. Thus, equation (4) can be further simplified as follows.

$$E_F = \hbar \nu_F \sqrt{\pi}\left(n_{res}^2 + \left(\alpha V_{CNP} \pm \beta\left(\frac{P}{q}\right)\right)^2\right)^{1/4}, \tag{5}$$

Equation (5) includes three unknown parameters: n_{res}, V_{CNP}, and β. As the ferroelectric polarization (P) can be precisely controlled by external gating, three equations corresponding to distinct polarization values ($P = +P_R$, 0, $-P_R$) can be obtained as follows.

$$E_{F1} = \hbar \nu_F \sqrt{\pi}\left(n_{res}^2 + \left(\alpha V_{CNP} - \beta\left(\frac{+P_R}{q}\right)\right)^2\right)^{1/4}, \tag{6}$$

$$E_{F2} = \hbar \nu_F \sqrt{\pi}\left(n_{res}^2 + (\alpha V_{CNP})^2\right)^{1/4}, \tag{7}$$

$$E_{F3} = \hbar \nu_F \sqrt{\pi}\left(n_{res}^2 + \left(\alpha V_{CNP} - \beta\left(\frac{-P_R}{q}\right)\right)^2\right)^{1/4}, \tag{8}$$

From the Fig. 1c and 1d in the main manuscript and the Fig. 4 and Note 2 in the supplementary information, it can be seen that the graphene used for the experiment was p-doped and $|\alpha V_{CNP}|$ was larger than $|\beta P_R/q|$. Thus, the inequality of $|E_{F3}| > |E_{F2}| > |E_{F1}|$ holds for our experiment. By solving the equations (6-8) with the inequality, analytic formulas for V_{CNP}, β, and n_{res} can be obtained as follows.

$$V_{CNP} = \frac{\left(\frac{E_{F3}}{\hbar v_F \sqrt{\pi}}\right)^4 - \left(\frac{E_{F1}}{\hbar v_F \sqrt{\pi}}\right)^4}{2\alpha\sqrt{2\left(\frac{E_{F3}}{\hbar v_F \sqrt{\pi}}\right)^4 - 4\left(\frac{E_{F2}}{\hbar v_F \sqrt{\pi}}\right)^4 + 2\left(\frac{E_{F1}}{\hbar v_F \sqrt{\pi}}\right)^4}}, \tag{9}$$

$$\beta = \frac{q\left[\left(\frac{E_{F3}}{\hbar v_F \sqrt{\pi}}\right)^4 - \left(\frac{E_{F1}}{\hbar v_F \sqrt{\pi}}\right)^4\right]}{4\alpha V_{CNP} P_R}, \tag{10}$$

$$n_{res} = \sqrt{\left(\frac{E_{F2}}{\hbar v_F \sqrt{\pi}}\right)^4 - (\alpha V_{CNP})^2}\ , \tag{11}$$

By inserting the values of Fermi levels (E_{F1} = -99 meV, E_{F2} = -291 meV, and E_{F3} = -409 meV) extracted from the FDTD simulations (along with the extracted momentum scattering time of τ ~ 29 fs), the unknown parameters were calculated as V_{CNP} = 267 V, β = 0.237, and n_{res} = 7.13 × 10^{11} cm^{-2}. The residual carrier concentration n_{res} is comparable to previously published values [4, 5]. As mentioned above, $|\alpha V_{CNP}|$ of 6.17 × 10^{12} cm^{-2} for our device was larger than $\beta P_R/q$ of 6.08 × 10^{12} cm^{-2}. Therefore, the memory metadevice always operates in the p-type regime of graphene.

By equation (5) with extracted parameters, the E_F of graphene is plotted as a function of V_G in Fig. 12. For ferroelectric modelling [6], the following equations were used.

$$P = P_S tanh\left(\frac{V \pm V_C}{2\delta}\right)\ , \tag{12}$$

$$\delta = V_C\left(log\frac{1+P_R/P_S}{1-P_R/P_S}\right)^{-1}\ , \tag{13}$$

Supplementary References

[1] Kim, W. Y. *et al.* Development of Manipulation Technology of Ferroelectric Polymer Film: Photo-lithographic Patterning and Multilayer Formation, *Microelectron. Eng.* **88,** 1576-1581 (2011)

[2] Kim, W. Y. *et al.* Fabrication and Characterization of Ferroelectric Poly (Vinylidene Fluoride – Trifluoroethylene) (P(VDF-TrFE)) Thin Film on Flexible Substrate by Detach-and-Transferring, *IEICE T. Electron.* **E95-C**, 860-864 (2012)

[3] Zheng, Y. *et al.* Graphene field-effect transistors with ferroelectric gating. *Phys. Rev. Lett.* **105,** 166602 (2010)

[4] Dawlaty, J. M. *et al.* Measurement of the optical absorption spectra of epitaxial graphene from terahertz to visible. *Appl. Phys. Lett.* **93,** 131905 (2008)

[5] Li, X. *et al.* Large-area synthesis of high-quality and uniform graphene films on copper foils. *Science* **324,** 1312-1314 (2009)

[6] Miller, S. L. *et al.* Device modelling of ferroelectric capacitors. *J. Appl. Phys.* **68,** 6463-6471 (1990)
